# Genes Related to Fat Metabolism in Pigs and Intramuscular Fat Content of Pork: A Focus on Nutrigenetics and Nutrigenomics

**DOI:** 10.3390/ani12020150

**Published:** 2022-01-08

**Authors:** Isaac Hyeladi Malgwi, Veronika Halas, Petra Grünvald, Stefano Schiavon, Ildikó Jócsák

**Affiliations:** 1 Department of Agronomy, Food, Natural Resources, Animals and Environment (DAFNAE), University of Padua, Viale dell’ Università 16, 35020 Padova, Italy; stefano.schiavon@unipd.it; 2 Department of Farm Animal Nutrition, Kaposvár Campus, Hungarian University of Agriculture and Life Sciences, Guba Sándor Utca 40, 7400 Kaposvár, Hungary; halas.veronika@uni-mate.hu (V.H.); grunvald.petra@uni-mate.hu (P.G.); 3 Institute of Agronomy, Kaposvár Campus, Hungarian University of Agriculture and Life Sciences, Guba Sándor Utca 40, 7400 Kaposvár, Hungary; jocsak.ildiko@uni-mate.hu

**Keywords:** epigenetics, fat metabolism, genes, intramuscular fat, nutrigenetics, nutrigenomics, pigs

## Abstract

**Simple Summary:**

The intramuscular fat (IMF) or marbling is an essential pork sensory quality that influences the preference of the consumers and premiums for pork. IMF is the streak of visible fat intermixed with the lean within a muscle fibre and determines sensorial qualities of pork such as flavour, tenderness and juiciness. Fat metabolism and IMF development are controlled by dietary nutrients, genes, and their metabolic pathways in the pig. Nutrigenetics explains how the genetic make-up of an individual pig influences the pig’s response to dietary nutrient intake. Differently, nutrigenomics is the analysis of how the entire genome of an individual pig is affected by dietary nutrient intake. The knowledge of nutrigenetics and nutrigenomics, when harmonized, is a powerful tool in estimating nutrient requirements for swine and programming dietary nutrient supply according to an individual pig’s genetic make-up. The current paper aimed to highlight the roles of nutrigenetics and nutrigenomics in elucidating the underlying mechanisms of fat metabolism and IMF deposition in pigs. This knowledge is essential in redefining nutritional intervention for swine production and the improvement of some economically important traits such as growth performance, backfat thickness, IMF accretion, disease resistance etc., in animals.

**Abstract:**

Fat metabolism and intramuscular fat (IMF) are qualitative traits in pigs whose development are influenced by several genes and metabolic pathways. Nutrigenetics and nutrigenomics offer prospects in estimating nutrients required by a pig. Application of these emerging fields in nutritional science provides an opportunity for matching nutrients based on the genetic make-up of the pig for trait improvements. Today, integration of high throughput “omics” technologies into nutritional genomic research has revealed many quantitative trait loci (QTLs) and single nucleotide polymorphisms (SNPs) for the mutation(s) of key genes directly or indirectly involved in fat metabolism and IMF deposition in pigs. Nutrient–gene interaction and the underlying molecular mechanisms involved in fatty acid synthesis and marbling in pigs is difficult to unravel. While existing knowledge on QTLs and SNPs of genes related to fat metabolism and IMF development is yet to be harmonized, the scientific explanations behind the nature of the existing correlation between the nutrients, the genes and the environment remain unclear, being inconclusive or lacking precision. This paper aimed to: (1) discuss nutrigenetics, nutrigenomics and epigenetic mechanisms controlling fat metabolism and IMF accretion in pigs; (2) highlight the potentials of these concepts in pig nutritional programming and research.

## 1. Introduction

The intramuscular fat (IMF) or marbling is an essential pork sensory quality that influences the preference of the consumers and premiums for pork. Marbling is the streak of visible fat intermixed with the lean within a muscle fibre which varies with the breed (genetics), age, sex, nutrition, muscle type and muscle location [1,2]. From an economic viewpoint, the pork industry is faced with increasing lean pig genotypes characterized by reduced IMF content which has a minimum range between 2.2% and 3.4% [3]. As such, strategies to optimize fat deposition traits in pigs have been extensively researched [4,5,6,7,8]. Improving the quality of the fatty acid profile and IMF content of pork is a major interest to swine nutritionists, breeders and geneticists for health and economic reasons [9]. This remains critical to the industry. Fat metabolism and marbling are multiplex traits regulated by several genes which are directly or indirectly involved in fatty acid metabolism, cell proliferation and differentiation [10,11,12]. An approach in unwinding the expression pattern of lipid metabolism genes and the molecular mechanisms behind IMF deposition is being researched [13,14,15,16,17].

Nutrigenetics and nutrigenomics are distinct fields providing a holistic approach in unravelling how nutrient intake affects the entire genome response and molecular mechanisms involved in fat deposition [18,19,20]. Nutrigenetics and nutrigenomics as fields of nutritional genomics research integrate computational systems biology (bioinformatics) with high-throughput functional genomic technologies (transcriptomics, proteomics, metabolomics and muscle biochemistry) in understanding how the cellular pathways and the entire genome respond to nutritional programming in farm animals [7,21,22,23,24]. Several factors such as the genetic make-up of the pig, sex, age, dietary micronutrients, etc., and environmental conditions, influence fat metabolism and phenotypic responses in pigs [15,16,17]. For instance, studies have evidenced that the combined effects of nutrients in the diet and environmental conditions could result in up-regulation/down-regulation of one gene which will then sway the response of other genes, and in turn, alter the expression of these genes [25]. Additionally, the relationship between mRNA expression of lipid metabolism genes and nutrient availability during transcription could be linear or quadratic and also depends on the ability of carrier proteins to recognize only one substance or group of similar substances in diets [25,26,27,28]. Furthermore, nutrients in the diet may be assembled at secondary metabolic pathways to alter substrate concentrations or act as ligands for transcription factors for genes involved in fatty acid metabolism [29,30]. Literature has suggested the existence of a genetic correlation between dietary nutrient intake and fat metabolism genes in pigs. [14,15,16,17]. In pigs, epigenetic mechanisms (DNA methylation and histone modification) are intermediaries influencing mechanisms of fat deposition and are sensitive to environmental factors and dietary nutrients [31,32]. Today, studies are evincing patterns of epigenetic mechanisms and molecular pathways that regulate gene expression (switching transcription on and off) in offspring, and the regulatory effects of messenger ribonucleic acids RNAs (mRNAs) and microRNAs (miRNAs) in fat and IMF depositions in pigs [31,32,33,34,35].

The underlying molecular mechanisms involved in fatty acid synthesis and marbling in pigs is difficult to unravel. Existing quantitative trait loci (QTL) for genes and their mutations in lipogenesis, disease susceptibility and the development of other traits in pigs are yet to be harmonized. Studies on the role(s) of epigenetic mechanisms in transgenerational effects of nutrition and environment in adipocyte differentiation and development of traits in pigs are lacking. To date, these gaps still exist in the literature. The scientific explanations behind the nature of the existing correlation between the nutrients in the diet and genes remain unclear, being inconclusive or lacking precision. This review aimed to: (1) discuss the roles of nutrigenetics, nutrigenomics and epigenetic mechanisms controlling fat metabolism and IMF accretion in pigs; (2) highlight the potential application of these concepts in pig nutritional research in nutritional intervention for swine production and the improvement of economically important traits in animals.

## 2. Introduction to Nutrigenetics and Nutrigenomics

It is important to clearly distinguish between nutrigenetics and nutrigenomics as these two distinct terms are often confused. For the purpose of intelligibility of scientific communication and reports in these domains, it is important to define certain words used herein. “Nutri” or nutrient refers to chemical compounds in a diet needed for cellular functions. Genetics is the study of individual genes, whereas genomics is the study of the entire genome (the whole of an organism’s genes, their interactions, and how they are affected by the environment). Therefore, we could infer that a common relationship between nutrigenetics and nutrigenomics is diet–gene interaction.

Verbatim definitions of nutrigenetics and nutrigenomics as expressed by different authors are quoted below:

*“Nutrigenetics is concerned with how genetic variation affects the interaction between these bioactive dietary components and the health and disease potential of individual persons while nutrigenomics is concerned with the effects of bioactive dietary components on the genome, proteome (the sum total of all proteins), and metabolome (the sum of all metabolites)” [36]. “Nutrigenetics focuses on the potential effects of single-nucleotide polymorphisms, copy number variants, epigenetic marks, and other genomic markers on the biological and behavioural responses to micronutrients, macronutrients, and calories whereas nutrigenomics has evolved to signify the field concerned by the investigation of the effects of nutrients on gene expression and related downstream molecular and biological events. Nutrigenomics will increasingly incorporate transcriptomics, proteomics, and metabolomics” [37]. “Nutrigenomics has evolved to signify the field concerned by the investigation of the effects of nutrients on gene expression and related downstream molecular and biological events while nutrigenomics will increasingly incorporate transcriptomics, proteomics, and metabolomics” [38]. “Nutrigenetics aims to understand how the genetic makeup of an individual coordinates the response to a diet while nutrigenomics offers a powerful and exciting approach to unravelling the effects of diet on health” [39]. “The term nutrigenetics refers to the impact of inherited traits on the response to a specific dietary pattern, functional food or supplement on a specific health outcome while the term nutrigenomics refers to the effect of diet on gene expression” [40]. “Nutrigenetics includes the study of individual differences at the genetic level that sways individual responses to diet. These individual differences may be at the level of single nucleotide polymorphisms rather than at the gene level while nutrigenomics comprises the analysis of the effect of nutrient intake on the whole genome (complete genetic make-up; including epigenetic changes), the proteome (the sum total of all proteins), and the metabolome (the sum of all metabolites)” [41]. “Nutrigenetics studies the influence of the genetic variations in the body promoted by the nutrients while nutrigenomics studies the influence of the nutrients on gene expression” [42].*

Each definition provided by the cited authors presents nutrigenetics and nutrigenomics as the science which integrates “omics” tools in providing insights into the nature of the interaction between inherited genes and nutrients in the diet. The importance of the application of nutrigenetics and nutrigenomics has since been utilized in human nutrition for understanding disease onsets and has been used to birth treatment options based on the concept of “individualized nutrition” [26]. In pigs, the combined effect of diets, genes, sex, age, environment, etc., on disease susceptibility, growth performance, fat metabolism and meat quality traits are starting to emerge. It could be hypothesized from Fench et al. [25] that just as in humans, the existence of differences in inherited genes affects nutrient bioavailability and metabolism in pigs regardless of breed differences.

## 3. Genes Involved in Fat Metabolism and IMF Accretion in Pigs

The post-genomic era has advanced the knowledge of genes that are associated with the molecular and genetic basis for fat deposition and IMF development in pigs. Studies have shown that most fat metabolism-related genes indirectly influence the IMF content of pork. However, their effects have shown variability with regards to muscle location and mechanisms of lipogenesis and adipogenesis [24]. Local pig breeds (such as Italian Landrace, local Basque, local Wujin, Mangalitsa, Meishan, etc.) present higher IMF content and better meat quality traits compared to modern breeds (e.g., Duoc–Iberian crosses, Large White breed, etc.). Higher expressions of genes and enzymes involved in fatty acid synthesis and lipid metabolism have shown to be the key drivers of the observable increase in IMF content of such local pig breeds [14,24].

Genes which are mostly implicated for their active role(s) in lipid metabolism and fatty acid synthesis in pigs and other animal species include: acetyl-CoA carboxylase alpha (*ACACA*), acyl-CoA oxidase 1 (*ACOX1*), acyl-CoA synthetase long-chain family member 3 (*ACSL3*), acyl-CoA synthetase short-chain family member 2 (*ACSS2*), adiponectin (*ADIPOQ*), adiponectin receptor 1 (*ADIPOR1*), 1-acylglycerol-3-phosphate o-acyltransferase 1 (*AGPAT1*), CCAAT/enhancer-binding proteins (*C/EBP*), alpha (*CEBPα*), CCAAT/enhancer-binding proteins (*C/EBP*), beta (*CEBPβ*), Catalase (*CAT*), diacylglycerol acyltransferase 1 (*DGAT1*), diacylglycerol acyltransferase 2 (*DGAT2*), fatty-acid-binding protein 3, muscle and heart (*FABP3* and *H-FABP*), fatty-acid-binding protein 4, adipocyte (*FABP4* and *A-FABP*), fatty acid synthase (*FASN*), leptin (*LEP*), leptin receptor (*LEPR*), lipase, hormone-sensitive (*LIPE* and *HSL*), lipoprotein lipase (*LPL*), peroxisome proliferator-activated receptor alpha and gamma (*PPARα* and *PPARγ*), retinoid X receptor gamma (*RXRγ*), solute carrier family 2 (facilitated glucose transporter) member 4 (*SLC2A4* and *GLUT4*) and sterol regulatory element-binding transcription factor 1 (*SREBF1* and *SREBP-1C*) [25].

### 3.1. Adipogenesis and Lipogenesis

**Figure 1:**
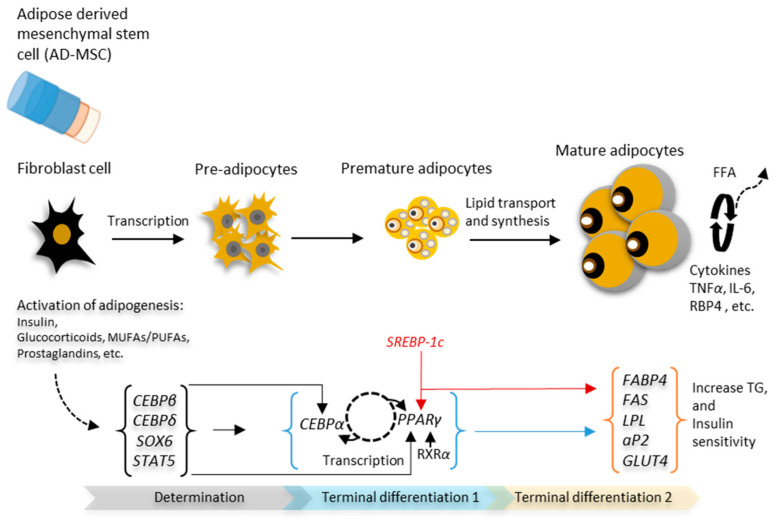
Schematic representation of adipocyte differentiation during adipogenesis. Adipocyte protein 2 = *aP2*; CCAAT/enhancer-binding protein = *CEBPβ* and *CEBPδ*; fatty-acid-binding protein = *FABP4*; glucose transporter type-4 = *GLUT4*; lipoprotein lipase = *LPL*; peroxisome proliferator-activated receptor gamma = *PPARγ*; retinoic X-receptor = *RXRα*; sterol regulatory element-binding protein-1c = *SREBP-1c*; tumor necrosis factor-alpha = *TNFα*.

Adipogenesis is a cell differentiation process where fibroblast-like preadipocytes develop into mature adipocytes regulated by the *PPARγ* gene, while the process of fatty acid and triglyceride synthesis is called lipogenesis. Both processes are regulated by different adipogenic and lipogenic genes, respectively [43,44]. Many authors have described the mechanisms controlling growth (increase in number and size; hyperplasia and hypertrophy, respectively), adipogenesis and lipogenesis [43,44,45,46]. For a polygenic trait such as fat metabolism, during transcription and adipogenesis, transcription factors bind specifically to the promoter region of their target genes and control their expression in different metabolic pathways [26]. In pigs, the determination and terminal differentiation stages of adipocyte differentiation occur in the adipose tissue. Conversely, in poultry, these stages of adipogenesis occur in the liver [9,43]. Adipogenesis is a consequence of the interaction between *PPARγ* with several different co-regulators involved in the control of the differentiation of fibroblast cells. At the determination stage, increased *CEBPβ* and *CEBPδ* activates *CEBPα* and *PPARγ*. *CEBPα* induces *PPARγ* expression as well as its expression. This cycle of interaction between *PPARγ* and *CEBPα* maintains increased levels of *PPARγ* and *CEBPα* and subsequently results in the start of adipocyte differentiation [43]. From examined literature [26,43,44,45,46], a simplified schematic representation of the process of adipose tissue development is presented in Figure 1.

### 3.2. The De Novo Fatty Acid (FA) Synthesis

**Figure 2:**
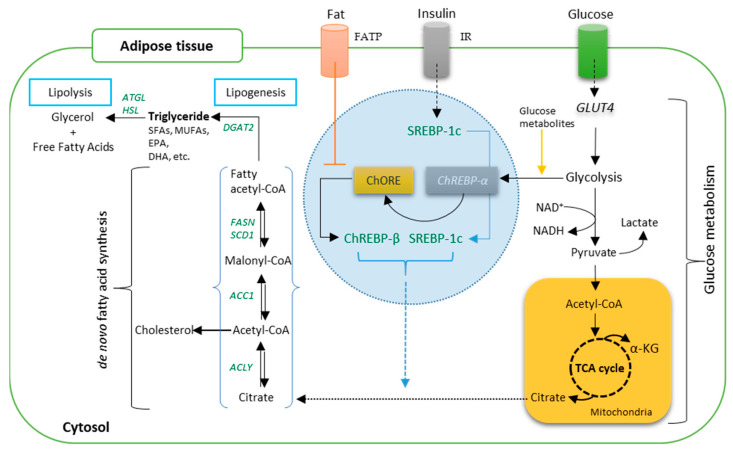
Schematic representation of de novo fatty acid (FA) synthesis from adipose tissue. ATP-citrate lyase = *ACLY*; acetyl-CoA carboxylases 1 = *ACC1*; carbohydrate response element-binding protein *α* and *βI* = *ChREBP-α* and *ChREBP-βI*; fatty acid transport protein-1 = FATP; fatty acid synthase = *FASN*; stearoyl-CoA desaturase-1 = *SCD1*; lipogenic transcription factor sterol regulatory element-binding protein-1 = SREBP-1; diacylglycerol O-acyltransferase homolog 2 = *DGAT2*; insulin receptor = IR; short-chain fatty acids = SFA; monounsaturated fatty acids = MUFAs; docosahexaenoic acid = DHA; Eicosapentaenoic acid = EPA.

During lipogenesis in the adipose tissue, glucose is converted into triglycerides through glycolysis and tricarboxylic acid (TCA) cycle, generating the energy required by the pig for metabolic activities [43,44,45]. However, this process varies between different breeds, fat depots and between the sexes. When glycolysis is initiated as a response mechanism to an increase in glucose or insulin, citrate is formed from the TCA cycle and used for de novo lipogenesis (de novo fatty acid synthesis). In response to carbohydrate intake, glucose is taken by adipocytes through insulin-stimulated *GLUT4* (see Figure 2). There are several published schematic representations of the pathways involved in *de novo* fatty acid synthesis [43,44,45,46,47,48,49]. A simplified pathway is shown in Figure 2.

Figure 2 shows the conversion of glucose to pyruvate through the cytosol of the cell tissue and transported into the mitochondria for further oxidation in the TCA cycle to produce citrate. In response to insulin secretion, the expression of *SREBP-1c* is initiated for adipocyte lipogenesis. The citrate generated from the TCA cycle is then exported back into the cytosol as a substrate for de novo lipogenesis which subsequently results in the release of acetyl-CoA by ACLY. *FASN* then converts malonyl-CoA to palmitate which becomes elongated to produce oleic, stearic and palmitic acid. The activation of *ChREBP-α* by glucose metabolites (generated during glycolysis) binds to promoter regions of *ACLY*, *ACC1*, *FASN*, *SCD1*, and *ChREBP-β* coding genes. Fatty acid synthesis is then promoted by the *ChREBP-β* sequel to activation of its target genes. However, fat intake blocks the expression of *ChREBP-β* and suppresses de novo lipogenesis [43,44,45].

Poklukar et al. [46] published a detailed review on the transcriptomic networks, hormones and enzymes modulating transcriptional regulation of adipogenesis in local and modern pig genotypes. Additionally, other studies have also revealed putative IMF accretion and fat metabolism-related genes [45,46,47,48,49], hormones, enzymes, transcription factors, and miRNAs [50,51,52] and their interaction with dietary nutrients [2,12,53,54] in pigs. Other findings evinced the possible association of genes influencing fat deposition and IMF accretion to the mitogen-activated protein kinase (MAPK) pathway regulating adipogenesis and lipogenesis [55,56]. However, studies on such mechanisms related to fat metabolism and pork quality traits, including IMF, are limited while existing few investigations remain elusive.

Active enzymes and their functional roles in fat metabolism and IMF include: hormone-sensitive lipase (LIPE) involved in IMF hydrolysis [57], acetyl-CoA carboxylase (ACC) which regulates the irreversible formation of malonyl-CoA from acetyl-CoA, fatty acid synthase (FAS) which regulates the synthesis of palmitate from acetyl-CoA and malonyl-CoA, stearoyl-CoA desaturase (SCD) that controls the transformation of monounsaturated fatty acids (MUFAs) from short-chain fatty acids (SFAs), and glucose-6-phosphate DH (G6PDH) and malic enzyme (ME) which generate nicotinamide adenine dinucleotide phosphate NADPH for reductive biosynthesis of fatty acids [46,58]. Main hormones such as insulin and glucocorticoids are reported to be involved in the regulation and initiation of adipocyte differentiation [59], depending on the existence of differentially methylated sites for genes involved in lipid metabolism and their associated pathways, as well as the muscle tissue location [46,60].

Some studies indicate the genes that could be considered as functional genetic markers and nutritional targets for individual nutrient-matching and dietary nutrient-based trait improvement strategies in pigs. These studies have shown how promising applications of “omics” based technologies are in nutritional genomics. A summary of the genes which are directly or indirectly involved in fat metabolism and IMF accretion in pigs are presented in Table 1.

### 3.3. Most Implicated Genes in Fat Metabolism and IMF Deposition in Pigs

Different studies have reported many genes that are associated with fat metabolism and IMF content in pig breeds. Nonetheless, when the whole-body fat depots of the pig are considered, it has been observed that variations exist between each fat depot and pig breed [62]. The genes that are mostly studied as key actors in adipogenesis, lipogenesis and IMF accretion in pigs are discussed below.

*PPAR* genes: Mainly, *PPARα* and *PPARγ* are a sub-family of the nuclear hormone receptor (*NHR*) super-family associated with metabolic pathways that are related to fat adipogenesis, lipogenesis, and gluconeogenesis [82,83,84]. *PPARα* and *PPARγ* are the most studied and implicated isoforms of the *PPARs* related to fat metabolism in pigs [71,85]. While *PPARα* is an important regulator for the transcription of genes that are involved in lipid metabolism, *PPARγ* principally regulates adipogenesis and promotes adipocyte differentiation and glucose homeostasis [86]. In newborn piglets, *PPARγ* expression is regulated by several transcription factors; however, its differential expression among piglets is yet to be established [85]. The gamma factor of the *PPARγ* is essential in the differentiation and maturation of preadipocytes and adipocytes, respectively, and it also induces the activation of fat cells through the *PPAR* transcription factor [71]. Higher concentrations of *PPARα* are found mainly in organs such as the liver while *PPARγ* is more concentrated in the adipose tissue of the longissimus dorsi muscle [86]. Interestingly, *PPARs* are activated by polyunsaturated fatty acids and their expressions vary between lean and fat pig genotypes [87].

*FABP* genes: Adipocyte and heart fatty-acid-binding proteins (*A-FABP* and *H-FABP*) are involved in fat metabolism and carry out intracellular transport of fatty acids from the cell membrane to sites of fatty acid oxidation [64,88]. The *H-FABP* (*FABP3*) gene is expressed predominantly in heart and skeletal muscle cells, while *A-FABP* (*FABP4*) is expressed almost exclusively in adipocytes [89]. Their expression tends to increase with the maturation of the longissimus dorsi muscle, thus affecting the expression of lipogenic genes [53,89]. Under the *FABP* class of genes, the *FABP3* and *FABP4* types are found to be associated with the marbling and IMF content of pork [65]. Studies have shown *FABP3* to be a strong genetic marker for IMF deposition and could independently influence IMF content and fatness traits in pigs [74,90]. In another study, *FABP3* expression was shown to be reduced in pigs with higher IMF and it is more strongly associated with the accretion of backfat when diets with low-fat contents are fed to pigs [66]. The expression of the porcine *A-FABP* (*FABP4*) gene varies between breeds. For example, its role in cell differentiation and IMF accretion is found to be more in Duroc pigs than in Meishan pigs [88]. The study of Chen et al. [89] reported a positive correlation between the *A-FABP* mRNA expression level and IMF content in Laiwu and Lulai Black pig populations. Despite this variability observed between breeds, *FABP4* has been proposed as a candidate gene in pig nutrigenomics applications due to its functional role in adipogenesis and increased IMF content [89,91].

*SCD* gene: Stearoyl-coenzyme A desaturase gene (*SCD*) is a functional gene that encodes an important enzyme stearoyl-CoA desaturase necessary for the conversion of saturated fatty acids (SFAs) into monounsaturated fatty acids (MUFAs) [92]. The *SCD* gene has been associated with the fatty acid composition of porcine longissimus dorsi muscle [79], and acts as an important regulator of the genetic mechanism of lipid deposition and fatty acid synthesis in pigs [77,82,92]. Additionally, it is involved in the *PPAR* signalling pathway and is important for meat quality traits in pigs [72]. The downward regulation in the expression of *SCD* gene was reported to be accompanied by an increase in the saturated fatty acid level in the adipose tissue [93], while up-regulation of *SCD* gene expression showed an increase in IMF content [72].

*LEP* (*LEPR*) gene: Porcine leptin and its receptor, *LEPR*, are known to be involved in food intake and energy homeostasis, and strongly affect the rate of IMF accretion. Its expression level tends to increase with age in pigs [67]. Generally, fatness is associated with leptin production and plasma level, thus, an increased expression of the *LEP* gene is expected in animals with increased fat deposition as has been observed in the fatty pig breeds [75]. *LEPR* is a candidate gene involved in fat metabolism, influencing not only IMF content but other pork quality traits such as moisture, cholesterol and flavour [66]. It has been recognized as one of the most functional genetic markers influencing growth and fat deposition in pigs [94]. As the IMF content tends to increase, Ros-Freixedes et al. [75] observed that the ratio of saturated fatty acids to polyunsaturated fatty acids (SFA: PUFA) tends to increase with more saturated fatty acids (SFA) and less polyunsaturated fatty acids (PUFA) in the porcine muscle [75]. *LEPR* gene expression controls the rate of IMF content and alters the fatty acid profile of the longissimus dorsi muscle.

*ACACA* and *FASN* genes: Acetyl-CoA carboxylase-α (*ACACA*) is a protein-coding gene while fatty acid synthase (*FAS*) is an enzyme encoded by the *FASN* genes. Both genes regulate the de novo synthesis of fatty acids from acetyl-coenzyme A and malonyl-co-enzyme A in the presence of NADPH [78,95]. Their expression levels also vary across breeds of pigs [78,95]. *ACACA* and *FASN* initiate the synthesis of fatty acids and saturated fatty acids during the early stages of lipid metabolism [46,78]. Studies have shown that the *FASN* gene is associated with IMF content and lipid metabolism pathways and is a candidate gene influencing fat traits in pigs [95,96]. However, Piórkowska et al. [78] recently reported that IMF content in Polish Landrace and Polish Large White pigs was influenced by a mutated *ACACA* gene. Zhao et al. [62] suggested that the mechanism of an increased rate of IMF deposition is related to a decrease in the rate of lipolysis and an increased rate of lipogenesis in fatty pigs. Such a mechanism is found to regulate the activity of *FASN* gene during anabolism, catabolism and fatty acid transportation [62]. The effect of *FASN* gene expression in IMF deposition in the porcine longissimus muscle is not clear; however, it was suggested to have a functional role as an enzyme of fat storage with several effects in subcutaneous adipose tissue and intramuscular fat tissue [62]. In Polish Large White pig breeds, the effect of the *FASN* gene is not largely detected on fat metabolism and IMF content [94]. Nonetheless, a recent longissimus dorsi transcriptome analysis confirmed that the *FASN* gene is key in lipid metabolism and highly associated with high IMF content in pigs [25,82].

*MSTN* or *GDF8* gene: The myostatin or growth differentiation factor 8 (*MSTN* or *GDF8*) gene belongs to the transforming growth factor-beta (*TGF-β*) super-family. It is responsible for double muscling in cattle and Belgian domestic pig breeds, as well as in *MSTN*-knockout pigs [97]. Although naturally occurring *MSTN* mutation is yet to be established in pigs [98], it is reported to be associated with reduced fat metabolism [79], and significantly lower IMF content in *MSTN* mutant mouse lines [99,100]. Inducing *MSTN* mutation in pigs could result in an increase in longissimus dorsi muscle area, better lean meat yield, reduced backfat and carcass fat content in pigs [100]. Despite its involvement in muscle development and pork quality characteristics, there is limited scientific evidence on the functional role of the porcine *GDF8* gene in fat metabolism and IMF accretion in pigs. This gap necessitates further research to understand how it influences pork fat metabolism, IMF deposition and other meat quality traits. A study [101] shows that *MSTN* knockout using CRISPR/Cas9-mediated genome editing with subsequent somatic cell nuclear transfer offers a promising possibility for genetic improvement of economically important traits in pigs. Ren et al. [79] demonstrated the active potential of *MSTN* in inhibiting the growth of muscles (double muscling) and acts via *myogenic transcription factor 2C* (*MEF2C*) which binds to the miR-222 promoter and suppress the translation of *SCD5* to affect fat deposition [79].

*SREBF-1* (*SREBP-1c*) gene: Sterol regulatory element-binding transcription factor-1c (*SREBF-1c*) was suggested to be an important lipogenic gene that has a critical role in the gene transcription mechanism and regulation of muscle fat deposition [62,102]. The role of *SREBF-1* in fat metabolism and IMF accretion remains contradictory between studies and could be breed dependent. The role of *SREBP-1c* in increasing lipogenesis and accompanied reduction of lipolysis in Wujin pigs is associated with increased adipocyte diameter, polyunsaturated fatty acid levels and IMF content [62]. Due to its regulatory role in muscle fat deposition during post-natal growth, it could be targeted as a gene marker for the genetic improvement of IMF in pigs [103]. While Chen et al. [103] reported a positive correlation between the expression of *SREBF-1* mRNA and IMF accretion in the longissimus dorsi muscle of pigs [103], Stachowiak et al. [104] found no association between *SREBF-1* gene transcript levels and fatty acid compositions in longissimus dorsi muscle and adipose tissue. Such differences require more investigation to understand the clear role of the *SREBF-1* gene in porcine fat metabolism and marbling.

## 4. QTL Regions and SNPs for Fat Metabolism and IMF Accretion in Pigs

Genome-wide association study (GWAS) has uncovered many key single nucleotide polymorphisms (SNPs or mutations) for genes and their quantitative trait loci (QTLs), sphingolipid signalling pathways, and enzyme co-factors related to fatness traits in pigs, [105,106,107,108]. However, it is yet unknown the gene (s) controlling mechanisms of IMF deposition in pigs. Pieces of literature have strongly suggested a difference in the gene expression and heritability (below 0.5%) for IMF deposition during muscle adipogenesis, myogenesis, lipogenesis and lipolysis, occurring at different stages of growth and development [69,107,108,109,110,111]. Certain genes are found to affect IMF deposition independent of backfat in pigs. For instance, Zhang et al. [112], revealed that QTL located on *Sus Scrofa* (SSC) 1 (167938652, 166363826, 164829874 and 167171587) and transducin-like enhancer of split 3 (*TLE3*), SMAD family member 6 (*SMAD6*), progestin and adipoQ receptor family member 5 (*PAQR5*) and integrin subunit alpha 11 (*ITGA11*) genes are associatd with IMF content accretion without affecting backfat in Duroc pigs. Such molecular markers are important in pig breeding programs targeted at IMF content improvement in pigs. Also, the applications of biological and dietary markers in marker-assisted selection for better fat deposition and IMF content are useful in pig nutrigenetic intervention [111].

Few QTLs associated with the *Sus Scrofa* chromosomes (SSC) 4, 6, 8, 13 and 14 have been reported to be more often involved with IMF deposition and fatty acid (SFAs and MUFAs) profiles in pigs [24]. The pig SSC14 and SSC6 QTLs have known regions for lipid metabolism and are related to *LEPR* and *SCD* genes with mutations or quantitative trait nucleotide (QTN) [93,106]. Earlier, QTL located on chromosome 4 (SSC4) was found to be responsible for the difference in fat deposition [106,113]. Today, about 778 QTLs related to different traits have been identified and documented in the pig QTL database, pigQTLdb (see https://www.animalgenome.org/cgi-bin/QTLdb/SS/index, accessed on 23 December 2021). Studies by Harper and Pethick [102] reported that the onset of marbling is located at chromosomal regions for QTL on chromosome 5 (SSC5), which is responsible for muscle growth and fat deposition. This QTL was genetically related to the *RARγ* gene which is involved in the transcription and expression of many other genes [114]. Later on, candidate genes associated with QTL on chromosome 6 (SSC6) were used to establish the functional role of the *RARγ* gene in fat deposition and marbling in pigs [115].

SNPs in pigs’ fat mass and obesity (*FTO*) gene are strongly associated with backfat and marbling and regulate average daily gain and lipid deposition [116]. Findings by Meadus and co-workers [117] revealed sire variability in terms of the IMF content of pork using SNP markers on chromosomes 5, 7, and 16. This implies that every sire is unique in terms of marbling genes [117]. Several chromosomal regions (QTLs) and molecular markers (SNPs) are now providing insights into specific candidate gene(s) controlling growth, nutrient uptake, disease resistance, meat quality traits and fat metabolism [93,105]. However, it remains a major challenge to nutritionally sway existing differentially methylated sites where genes involved in lipid metabolism are found [118].

Transcriptome analysis has deepened our scientific knowledge of the molecular pathways and genetic basis of fat metabolism and IMF accretion in pigs [12,94,119]. To this end, there is clear evidence that the use of nutrient-gene biomarkers is a crucial fingerprint for accurately elucidating the genetic and nutritional regulation of fat metabolism. Potential QTLs of complex traits and functional genes related to muscle growth, fat and IMF deposition, and many putative genes involved in the mechanism of fat distribution and marbling in pigs are becoming available [47,114,120,121]. Despite the far-reaching pieces of evidence from literature, the application of DNA-specific markers in simultaneously enhancing fat deposition and IMF content of pork without altering other carcass traits remains difficult to achieve. In addition, the precision of mapping the existing gene markers in terms of selection across breed populations for genetic variation remains limited [75,117].

## 5. Epigenetic Mechanisms: Role of mRNAs, miRNAs, DNA Methylation and Histone Modification in Fat Metabolism

Genome-wide high throughput DNA analysis was recently developed to profile the human and animal genomes [122,123]. Literature is starting to evince significant epigenetic responses associated with fat deposition, mainly the role of DNA methylation in the regulation of gene activities, and how genes are expressed in pigs and other species (cow, chicken, etc.) [31,32,33]. Also, epigenetic memory is reported to be associated with some DNA methylation patterns which results in heritable phenotypic responses [124]. Epigenetics is the basis for heritable changes in gene expression without altering the original genetic code or DNA sequence itself [125]. It is the beginning of cell differentiation processes through which genes are turned “on” and “off” or silenced [33] and is influenced by environment and nutrition [34], whereas epigenomics is the analysis of epigenetic responses of genes in the entire epigenome chemical compounds and proteins that can attach to DNA during gene expression [117].

The effects of epigenetic mechanisms in the fat metabolism process are controlled by the transcriptional roles of miRNAs in binding to protein-coding genes, DNA methylation, and histone modification [124,125]. Epigenetic studies have revealed variability in differential DNA methylation patterns of lean and fat pigs [32]. Many genes regulated by differentially methylated promoters were implicated in lipid metabolism, sensory and olfactory processes, and ATPase activity [32]. In addition, polygenic trait effects related to IMF deposition and fat metabolism as well as their degree of heritability are controlled/regulated by epigenetic modifications [119,126]. The role of epigenetics in fat metabolism is becoming clearer as studies are uncovering the underlying pattern of expression of coding and non-coding genes as well as the functional role(s) of mRNA and miRNA during adipocyte and myocyte cell differentiation [125]. Thus, it is relevant to take into cognizance the important roles that epigenetics is playing in how pigs express phenotypic traits in response to nutrient intake.

### 5.1. Role of Messenger and Micro RNAs (mRNAs and miRNAs)

During DNA transcription and translation, the enzyme RNA polymerase catalyzes DNA base-pairing, which is regulated by miRNAs to produce a pre-mRNA transcript that is further processed into an mRNA molecule (a single-stranded copy of the gene). The mRNA is “read” based on the genetic code which relates the DNA sequence to the amino acid sequence in proteins (polypeptides) encoded by the original gene [127,128]. miRNA-mediated events include: translational repression, mRNA decay, RNA-binding protein inactivation, protein synthesis [129] and fatty acid metabolism through related pathways [62]. The literature suggests the indispensable role of miRNA in fat deposition and adipocyte differentiation [130,131]. Additionally, the use of miRNA sequence in investigating IMF content-related genes is uncovering differentially expressed genes (DEGs) associated with muscle growth and lipid deposition in pigs [56]. MiRNAs have the potential to down-regulate gene expression by blocking mRNA translation of certain genes. Their structure, synthesis and action in adipogenesis and their strong regulatory roles in animals have been extensively reviewed [127,128,129,130,131]. Mobuchon et al. [132] reported two miRNAs (miR-142-5p and miR-20a-5p) associated with *PPARα*, *PPARγ*, *ELOVL6* and *ACATI1* genes which are involved in nutrient-gene regulation mechanisms of cell proliferation, cell differentiation and lipid metabolism [77,132]. Furthermore, miRNAs in adipose and muscle tissue whose target genes are associated mainly with signalling pathways rather than metabolic and biosynthetic processes have been detected in various pig breeds [133,134]. While the behaviour of miRNAs tends to be dissimilar between breeds, their expression pattern also varies with age [133] and cell differentiation, such as osteogenesis, myogenesis, adipogenesis, etc. [133,134,135,136,137,138]. It has been established that even when isolated from the same tissue but different animal breeds, miRNAs’ differentially expressed gene profiles tend to be breed-specific [139]. Many studies have confirmed their involvement in myogenesis and adipogenesis by altering the expression of their target genes and proteins [52,131,140,141]. Wang et al. [77] reported the mechanism of lipid deposition from a transcriptome profile of pig muscle tissues. Their results revealed *CAV2*, *MYOZ2*, *FRZB*, miR-29b, miR-122, miR-145-5p and miR-let-7c as key genes and miRNAs, respectively, regulating muscle growth while *FASN*, *SCD*, *ADORA1*, miR-4332, miR-182, miR-92b-3p, miR-let-7a and miR-let-7e were key genes and miRNAs, respectively, involved in the regulation of lipid deposition in pigs. miRNAs’ involvement with mitogen-activated protein kinase (MAPK) cascades, a key signalling pathway that regulates a wide variety of cellular processes including cell proliferation, differentiation, apoptosis, and stress responses, have been documented [77]. The knowledge on the potential transcriptomic roles of such ribonucleic acids is changing approaches to trait improvement and is providing more information on epigenomic modifications associated with phenotypic variability in pigs [142,143].

### 5.2. DNA Methylation and Histone Modification in Fat Metabolism

DNA methylation is a biochemical gene modification process that determines gene expression patterns or “gene silencing” (regulating the turning “on” and “off” of some genes) related to the metabolic synthesis of fats. Histone modification involves histone acetylation, regulated by histone acetyltransferases (HATs), and deacetylation, on specific lysine residues regulated by histone deacetylases (HDACs) [144]. Gene expression involving the interaction of HATs, HDACs and histones can activate or repress gene transcription such that histone acetylation unlocks and activates chromatin, while chromatin becomes transcriptionally silent through deacetylation of histones and DNA methylation [144]. However, it is yet to be proven the clear role of DNA methylation and histone modification mechanisms in fat metabolism.

**Figure 3:**
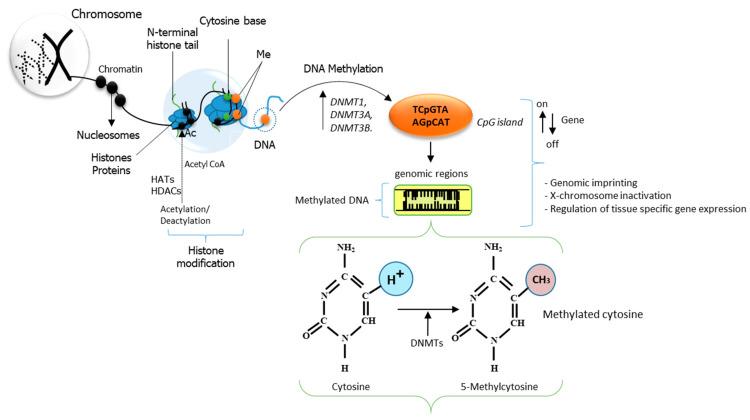
Epigenetic modifications of chromatin by histone modification and DNA methylation of cytosine nucleotides on the 5th carbon of the cytosine base at the CpG site.

Nutrition and environmental factors have a significant effect on DNA methylation, leading to an increase in the expression of genes related to production performance, disease and meat quality traits. DNA methylation is regulated by DNA-methyl-transferase enzymes (DNMTs) and methyl-CpG-binding domain proteins (MBDs) during gene expression in mammals [145,146,147]. Specifically, DNMT1 maintains DNA replication and cell division while DNMT3A and DNMT3B maintain de novo methylation during early development. A diagram showing the pathway involved in DNA methylation and histone modification is shown in Figure 3.

Histone modification alters gene expression through mechanisms of HATs’ and HDACs’ functions during acetylation of histones at their lysine residue sites. Histone modification begins with the addition of an acetyl group (Ac) by acetyl CoA followed by HATs regulated acetylation. HDACs serve as catalysts for the hydrolytic removal of the acetyl groups from histone (Figure 3). When this mechanism is altered, mutation and disease or trait progression are observed. DNMT1, DNMT3A and DNMT3B initiate and maintain CpG methylation across the genome by either blocking or allowing binding of proteins associated with methyl-CPG-binding sites [148]. Such sites are genomic regions where cytosine is separated from guanine by just a phosphate group (CpG islands) in a linear sequence of a base in the direction of 5′ → 3′ [149,150,151]. The effects of cytosine methylation within the base sequence of a gene include processes involving genomic imprinting, X-chromosome inactivation, suppression of repetitive elements, lipogenesis, and carcinogenesis [148]. DNMT1 has a significant regulatory effect on genes at the CpG-binding sites. Studies have shown that when it binds at CpG to the *SREBP1* gene, it down-regulates the activity of *SREBP1* while an unmethylated promoter exerts an opposite effect by up-regulating the activity of the *SREB1* gene during adipogenesis [152]. Another mode of action of DNMT1 shows that it regulates adipogenesis by promoting differentiation at an early stage while inhibiting lipogenesis at the late stage of preadipocyte differentiation [153].

Studies have shown that methylating dietary micronutrients elicited differential expressions of genes involved in lipid metabolism, and later, gene repression of certain housekeeping genes [23]. Qimuge and others [119] demonstrated that DNMT3A increased proliferation and inhibited the differentiation of intramuscular preadipocytes by decreasing the expression of cyclin-dependent kinase inhibitor 1A (*p21* also known as *CDKN1A*), and down-regulated the levels of *PPARγ*, *SREBP-1c*, and *FABP4* through the methylation of *PPARγ* promoter [119]. The study of Stachecka et al. [153] showed that the onset of adipogenesis elicited an increase in transcript level of *DNMT1* gene followed by a decrease, while *DNMT3A* and *DNMT3B* gene transcripts increase during the in vitro differentiation. This in vitro investigation on differentiation of mesenchymal stem cells (AD-MSC) into adipocytes established how the expression of DNMT transcripts proceed in the AD-MSC and bone marrow tissue (BM-MSC) [153]. Today, chromatin regulators can be targeted to regulate and control gene expression [147]. When combined with other nanobodies, DNMT3A have the potential to enhance gene silencing speed and epigenetic memory [147].

## 6. Nutritional Genomics in Pigs

### 6.1. Nutrigenetics and Nutrigenomics

**Figure 4:**
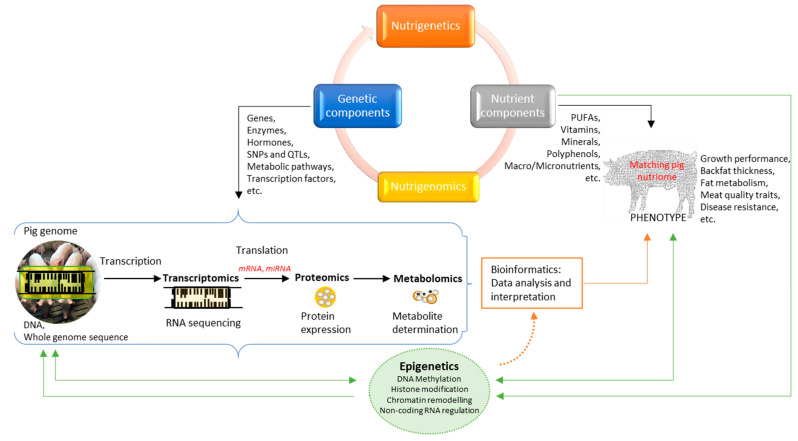
The schematic workflow chart in nutrigenetics, nutrigenomics, and epigenetics science.

While nutrigenetics shows the variation in DNA sequence in response to dietary nutrients, nutrigenomics deals with the roles of dietary nutrients in gene expression and/or structure [154]. Nutrigenetics deals with how the genetic predisposition of an individual pig controls its responses to dietary nutrients, whereas nutrigenomics deals with the effect of nutrient intake on the whole genome (complete genetic make-up, including epigenetic changes), transcriptomics (RNA transcripts that are produced by the genome), proteomics (proteins produced in an organism which changes from cell to cell and changes over time), and the metabolome (detailed characterization of metabolic phenotypes) of the pig [28,41]. Both nutrigenetics and nutrigenomics encompass the tenets of nutritional genomics. The inter-relationship between nutrigenetics, nutrigenomics and epigenetics is presented in Figure 4.

Since the completion of the human genome project, nutritional genomics emerged as a nutritional science that deals with nutrition, genome and health in understanding the genetic and nutritional basis of disease and ageing in humans [26,30]. Today, it has found enormous applicability in the field of animal nutrition research as well. Nutritional genomics offers the possibility to elucidate complex mechanisms of gene–nutrient interaction and the environment on the entire genome. The use of high-throughput DNA-based “omics” technologies and system biology is defining a new post-genomic era in nutritional genomics of animals (Figure 4). Nutrients can be matched more accurately with inherited genes to harmonize metabolic functions and improve health and economically important traits in animals [26]. Loor et al. [155] reported a summary of how the application of nutrigenetics and nutrigenomics in animal nutrition is promising in disentangling the complexities associated with interactions between nutrients, physiological status and cellular functions of dairy cows, pigs, and poultry. In addition, biological and nutritional pathways related mainly to fat metabolism have confirmed that matching nutriome (nutrient intake combination) in pigs to enhance cellular metabolic functions and desired genetic responses in pigs can be successful [45,59,60].

The main goals of nutritional genomics as summarized by Kaput and Rodriguez [30] include: (i) nutrients in the diet can alter the genome, either directly or indirectly; (ii) dietary nutrients and bioactive compounds have the potential to be “risk factors” for disease; (iii) some diet-regulated genes (and their normal, common variants) are likely to play a role in the onset, incidence, progression, and/or severity of diseases; (iv) the degree to which diet influences the balance between health and disease states may depend on an individual’s genetic makeup; and (v) disease can be cured or treated through a dietary intervention based on knowledge of nutritional requirements, nutritional status, and genotype (i.e., “individualized nutrition”).

Translating these five goals into disease and trait improvements in pigs has a wide range of applications in swine nutrition and could result in better phenotypic responses in a breeding program.

### 6.2. Impact of Dietary Nutrient Supply on Some Genes Related to Fat Metabolism and IMF Deposition in Pigs

The functional role of amino acids in muscle or adipose tissue content and gene expression have high applicability during nutrient intake combination. The impact of reduced feed intake resulted in an increased expression of *GLUT1* and *GLUT4* mRNA in the skeletal muscle of growing pigs [45]. Studies have shown that amino acids such as methionine, lysine, histidine, isoleucine, leucine, phenylalanine, threonine, tryptophan, and valine are essential in several metabolic pathways [35,156,157]. However, establishing their individual effects on gene responses remains a challenge due to data limitations and the complex variability between pigs’ genetics, environment and the quality and quantity of the nutrients in a given diet [17].

#### 6.2.1. Impact of Dietary Crude Protein Supply

Protein, fat and micro/macro-nutrient supplementation have been proposed as nutritional interventions applied during different growth and developmental stages of the animal (prenatal, neonatal, or post-natal) [158,159]. To elucidate the regulatory mechanisms of dietary protein levels on gene expression related to lipid metabolism, the study conducted by Zhao et al. [53] showed that high dietary protein supply at 18% CP significantly reduced expressions of mRNA, enzyme activities and expression levels of key fat and marbling genes in pigs. They demonstrated the effect of increasing body weight from 30 kg to 60 kg to 100 kg by feeding pigs with high or low protein diets. In the same study, gene expression was reduced at 60 kg and 100 kg with high protein dietary feeding. *ACC, FAS, SREBP-1c* and *PPARγ* expressions and enzyme activities of *A-FABP, LPL*, carnitine palmitoyltransferase 1B (*CPT-1B*), *PPARγ* and *SREBP-1c*, were promoted at 60 kg [53]. To achieve a significant effect on growth, body composition and gene expression patterns in the skeletal muscle of pig offspring, the best stage for applying nutritional intervention is suggested to be at gestation period and early life [160,161,162]. However, caution is needed as reducing protein supply in diets of gestating sows could impair fetal development as well as piglets’ life post-partum. Another study showed that dietary supplementation with alpha-ketoglutarate (AKG) increased the expression level of mRNA of *FABP4* and *FASN* genes during low dietary protein feeding of growing pigs at 44 ± 1 d of age (11.96 ± 0.18 kg BW) [163]. The number of adipocytes in longissimus dorsi and IMF content tends to increase following energy and protein feed restriction during the suckling stage in young piglets [163].

#### 6.2.2. Effect of Lysine, Methionine, Vitamin A, Micro/Macro-Nutrients

Lysine is an essential amino acid in pigs. A low supply of lysine in the diet of heavy finishing pigs alters the functional role of transcription factors such as *PPARγ*, *SREBF1* and adipocyte *FABP-4* [45]. Earlier studies by Katsumata et al. [162] have shown that reduced intake of lysine promotes the IMF deposition in the longissimus dorsi of finishing gilts by up-regulating the expression of the *PPARγ* gene [162]. Similarly, when six (6) week old pigs were fed the diet of three (3) week old piglets, *PPARγ* and *GLUT4* mRNA expression were upregulated following low dietary lysine supply in the longissimus dorsi and muscle rhomboideus of the pigs [164,165]. The mRNA expression of *GLUT4* was found to be higher in longissimus dorsi muscle of pigs fed a low dietary threonine [166].

In general, altering the level of dietary lysine regardless of the physiological status of the pig could have a huge nutrigenetic impact. Studies showed that a 0.78% lysine supply resulted in higher IMF content in growing pigs [167]. Methionine (formyl-methionine), arginine and lysine are the first three amino acids incorporated into any new protein during gene sequence determination [168,169,170]. Other nutrients such as α-linolenic acid have been shown to influence and alter expressions of *SREBP-1c* in the liver and 2,4-dienoyl CoA reductase 2 (*DECR2*) gene in the longissimus dorsi muscle [171]. Conversely, dietary lysine restriction (diets low in lysine: energy ratio) evinced better marbling and fat deposition rate during the growing-finishing period in lean pig genotypes [172,173]. The results of Schiavon et al. [173] indicated that reduced dietary crude protein supply resulted in better IMF content and fatty acid composition in heavy pigs [173,174]. Studies on the excess supply of lysine are scarce and this necessitates more studies to find out the effect of excess lysine supply on gene expression in pigs.

In the case of vitamin A (retinoid) supplementation, the effect of nutrient–nutrient interaction with vitamin A and its impact on nutrient bioavailability (absorption and utilization) related to fat metabolism and IMF accretion is still unclear. However, activation of the *PPARs* signalling pathway, *RAR* and *RXR*, using vitamin A (retinoid) promotes the process of fat metabolism [101]. When included in diet at 100,000 IU/kg, retinoid increased IMF content [21,168]. On the other hand, when retinoid was not added to the diet (at 0 IU/kg), no effect on IMF or fat content of the longissimus dorsi muscle was observed but a reduction in the expression of *PPARα* gene occurred [22].

Micronutrients influence the pattern of expression of several genes in pigs. They can modulate signalling pathways of genes and their regulatory elements during growth and development [161,175,176]. Additionally, dietary fatty acids have a vital regulatory effect on DNA receptors and enzymes during DNA transcription and translation [177,178]. Wang et al. [178] opined that when pigs are fed a low protein diet at growth-finishing stages, a direct relationship with higher expression of intramuscular lipogenic genes and decline in expression of a lipolytic gene is achieved. Another study by Kloareg et al. [179] showed the impact of feeding pigs with a diet containing 15 g/kg soyabean oil and 44 g/kg fat on body fat distribution of pigs. The pigs in the experiment were serially sacrificed between 90 and 150 kg. These pigs evidenced that 0.31 and 0.40 of the digested n-6 and n-3 FA were deposited, respectively, while about 1/3 of the n-3 supply that was deposited resulted from the conversion of 18:3 to other metabolites (i.e., EPA, docosapentaenoic acid and DHA). The study indicated that lipogenic and lipolytic activities change with increasing body weight, while in another study, the average whole-body fatty acid composition varies with tissue but remains constant during the finishing period of pigs [179].

The application of nutritional genomics in fine-tuning dietary nutrients to alter gene expression in pigs would no doubt lead to improvements in economically heritable traits, production performance, health and disease management [58,160]. Scanning an entire genome for the regions of increased or decreased copy number, or differentially methylated sequence will offer animal nutritionists unlimited possibilities to optimize feeding and meat quality traits (as IMF) in pigs. It can also mitigate pet and livestock disease. In addition to understanding the nature of gene–nutrient and environment interaction, research in the future could consider these unanswered questions:

(i) How can nutrients be matched to an individual pig’s genetic predisposition especially when dealing with the same genes controlling desired/undesired phenotypic traits in pigs?
(ii) How can we quantitatively define nutrient requirements in swine using an individual gene or whole-genome data to initiate an optimal metabolic or trait response?
(iii) How can we fine-tune nutrients and bioactive compounds in a diet to ensure the heritability of genes related to production performance (meat and milk quality), metabolism and genome stability?
(iv) How do we deal with genes capable of controlling different traits that are functionally interdependent such that altering one could lead to a responsive effect in another one?
(v) How can we harmonize nutritional genomic information in modulating genes and their transcriptional factors and subsequently match them with reference dietary nutrients to alter epigenetic response in pigs?

Thus far, from the literature, we can accurately map the genetic, physiological and nutritional regulatory pathways involved in many cellular functions such as molecular mechanisms of fat and IMF accretion in pigs. This has made the impact of individual dietary nutrients on the whole genome less elusive. Soon, harmonizing the existing knowledge of nutritional genomics might be the major tool for precise estimations of nutrient requirements of pigs with different physiological statuses, age, sex and breed for fat metabolism and other trait improvements (such as growth performance, backfat thickness, IMF accretion, disease resistance, etc.) in pigs and other livestock species.

## 7. Conclusions

Different studies have reported and confirmed a number of QTLs, SNPs, and mRNAs and miRNAs involved in molecular mechanisms of fat metabolism and IMF deposition in pigs. The main focus earlier was on the identification of single genes involved in the regulation of fatty acid synthesis and IMF deposition in pigs, but later, it was revealed that epigenetic factors and processes are also influential in this field. This might provide more significance of external factors, such as nutritional properties of feed, nutrients and dietary bioactive substances whose levels in the diet can be difficult to control, in addition to environmental factors.

The science of nutrigenetics, nutrigenomics and epigenetic mechanisms are efficient and precise in defining changes in gene sequences that predispose individual pig breeds to respond in a certain way in terms of performance, meat and milk quality as well as health and disease detection. As a result, it is possible to measure nutritional effects towards fine-tuning gene expressions and regulating genome responses in pigs, to optimize growth performance, backfat thickness, IMF deposition, disease resistance and meat quality traits. However, the question remains: how prepared are we to integrate this science as a tool in animal nutrition and swine feeding?

## Figures and Tables

**Table 1:** A list of genes related to fat metabolism and IMF deposition in pigs.

Study	Gene Name	Breed	Tissue	Sampling Age (d) or Body Weight (kg)	Trait
[60]	*FABP4, FASN*	Chinese local and Large White	LD, L	150 d	IMF
[61]	*ADIPOQ, PPARG, LIPE, CIDEC, PLIN1, CIDEA*, and *FABP4*	Purebred Duroc	LD	108 kg	IMF
[62]	*ATGL, FAS, HSL, CPT-1B, SREBP-1c, SCD, A-FABP* and *H-FABP*	Wujin and Landrace	LD	100 kg	IMF
[63]	*RAD9A, IGF2R, SCAP, TCAP, SMYD1, PFKM, DGAT1, GPS2, IGF1, MAPK8, FABP, FABP5, LEPR, UCP3, APOF*, and *FASN*	Landrace and Songliao Black sows	SF, LD, L	100 kg	Fat deposition
[64]	*H-FABP* and *LEPR*	Duroc, Pietrain, Puławska, Polish Large White (PLW), and Polish Landrace (PL)	LD, SMM, L	Slaughter at 6 age groups 60-, 90-, 120-, 150-, 180- and 210-d-old pig	Fat deposition and IMF
[65]	*FABP3* and *LEPR*	Duroc, Pietrain, Puławska, Polish Large White (PLW) and Polish Landrace (PL)	LD	100 kg	Fatty acid metabolism and IMF levels
[66]	*FABP3* and *LEPR*	Korean native pig and Yorkshire crossed animals.	LD	90–100 kg	IMF
[67]	*H-FABP* and *MASTR*	Large White	BL	95–105 kg	IMF
[68]	*PRKAG3*	Large White X Duroc X Pietrain	SM	110 kg	IMF
[69]	*EEF1A2, FABP3, LDLR, OBSCN, PDHB, TRDN* and *RYR1*	Landrace X Large White X Pietrain	LD	30, 60, 90 and 120 kg	IMF
[70]	*IGF2*	Large White, Polish Landrace and Puławska pigs	BL	100 kg	IMF
[71]	*PPARG* and *ADRP*	Laiwu, Lulai Black, and Large Whites	LD	114 kg	Fat deposition and IMF
[72]	*PPARA, PPARG, SCD* and *PCK2*	Shanzhu X Duroc commercial crossbreds	LD	90 kg	Lipid deposition and IMF
[73]	*BMPER* promoter	Duroc X Large White X Yorkshire	LD	-	IMF
[74]	*FABP3* promoter	Large White X Landrace background X Pietrain	LTL, SMM, BL	-	IMF
[75]	*SCD* and *LEPR*	Duroc	GM, LD	128 kg	IMF and fatty acid composition
[76]	*FASN* and *LIPE*	Jinhua and Landrace	SA	Slaughtered at 35, 80 and 125 days of age	IMF
[77]	*CAV2, MYOZ2, FRZB, FASN, SCD, ESR1*, and *ADORA1*,	Chinese Diannan Small-ear pig, Tibetan, Landrace and Yorkshire	LD	-	Lipid deposition and muscle growth
[78]	*SCD, ACACA*, and *FASN*	Puławska, Polish Large White and Polish Landrace	LD, BL	100 kg	IMF and lipid metabolism
[79]	*MSTN*	MSTN-knockout (KO) cloned Meishan	SF, BL	70 kg	Fatty acid metabolism
[80]	*FGF2*	Italian Large White	SMM	150 kg	IMF
[81]	*FABP3, LIPE, IGF1, IGF2, LEP, LEPR, MC4R, PHKG1, RETN, RYR1, SCD*, and *UBE3C*	Chinese Shuai pigs	LD	80–90 kg	IMF
[82]	*FASN, SCD, ELOVL6, DGAT2, PLIN1, CIDEC*, and *ADIPOQ*	Iberian	LD	165 kg	Lipid metabolism and higher content of IMF

BL = blood; GM = gluteus medius; L = liver; LD = longissimus dorsi; SA = subcutaneous adipose; SF = subcutaneous fat; SM = skeletal muscle; SMM = semimembranosus muscle; LTL = longissimus thoracis et lumborum.

## Data Availability

Not applicable.
